# Dissociated vacancies and screw dislocations in MgO and UO_2_: atomistic modeling and linear elasticity analysis

**DOI:** 10.1038/s41598-019-42926-z

**Published:** 2019-04-24

**Authors:** Xiang-Yang Liu, Enrique Martinez, Blas P. Uberuaga

**Affiliations:** Materials Science and Technology Division, Los Alamos National Laboratory, Los Alamos, New Mexico 87545 USA

**Keywords:** Atomistic models, Ceramics

## Abstract

Understanding the effect of dislocations on the mass transport in ionic ceramics is important for understanding the behavior of these materials in a variety of contexts. In particular, the dissociated nature of vacancies at screw dislocations, or more generally, at a wide range of low-angle twist grain-boundaries, has ramifications for the mechanism of defect migration and thus mass transport at these microstructural features. In this paper, a systematic study of the dissociated vacancies at screw dislocations in MgO is carried out. The important role of stress migration in the atomistic modeling study is identified. Another aspect of the current work is a rigorous treatment of the linear elasticity model. As a result, good agreement between the atomistic modeling results and the linear elasticity model is obtained. Furthermore, we demonstrate that the proposed vacancy dissociation mechanism can also be extended to more complicated ionic ceramics such as UO_2_, highlighting the generality of the mechanism.

## Introduction

Microstructural features in ionic ceramics have great influence on the ionic conductivities of such materials, which are tightly related to the mass transport of ions^1–8^. These microstructural features include interfaces, grain-boundaries, and dislocations. Among these microstructural features, dislocations are not only isolated defects themselves, but can also form the basic structural unit of other microstructural features such as low angle grain-boundaries^9^ or misfit dislocation networks of semi-coherent interfaces^10^. In addition, defect-dislocation interactions can also have important impact on the plasticity of materials. Thus, understanding the effect of dislocations on mass transport in ionic ceramics is important for understanding the behavior of these materials in a variety of contexts.

In the early 60s, Thomson and Balluffi^11,12^ suggested that vacancies could dissociate at screw dislocations in ionic materials. It was suggested that a charged defect such as a vacancy could decompose into four charged jogs and kinks along a screw dislocation, driven by a reduction of the Coulomb energy^9,12^. This mechanism was proposed to explain experimental evidence that in materials such as MgO, dislocations can form helical structures^13^. The proposed mechanism, i.e., the dissociated nature of vacancies at screw dislocations, or more generally, a wide range of low-angle twist grain-boundaries, has ramifications for the mechanism of defect migration and thus mass transport at these microstructural features^14^.

Only recently, this mechanism, now over 50 years old, was directly validated by atomistic simulations^14^, although a simpler picture (pair of jogs) was observed in the atomistic simulations. That work also developed an analytical model of the behavior of dissociated vacancies at dislocations, based on elasticity and electrostatics. The agreement between the model and the atomistic calculations in ionic rocksalt compounds (MgO, BaO, and NaCl) was found to be only qualitative, reproducing the basic trend but not the actual magnitude of the interactions. In the current work, we present a more systematic study of the problem. By focusing on MgO, we identify the important role of stress migration (as defined later) in the atomistic modeling study, which was neglected in our previous work^14^. Another aspect of the current work is a more rigorous treatment of the linear elasticity model, correcting an error in our previous work^14^. As a result, a much better agreement between the current atomistic modeling results and a revised linear elasticity model is obtained. Furthermore, we demonstrate that the proposed mechanism by Thomson and Balluffi^11,12^ can also be extended to more complicated crystal structures such as UO_2_, highlighting the generality of the mechanism.

## Results

### Atomistic modeling of MgO screw dislocations

MgO has a relatively simple rocksalt (B1 type) crystal structure with simple ionic bonding. In the B1 structure, cations and anions sit on two inter-penetrating fcc lattices. The preferred slip system is a_0_/2〈1 1 0〉$\{\bar{1}10\}$, both observed experimentally^15^ and predicted by density functional theory (DFT) calculations^16^, where a_0_ is the lattice constant. Comparison between DFT and a pairwise-potential of the Buckingham form of the 1/2〈1 1 0〉 screw dislocation core shows a remarkable agreement^17^.

The screw dislocations are modeled in a periodically repeating supercell containing a dislocation dipole, with + or −a_0_/2〈1 1 0〉 Burgers vector. The dislocation lines are arranged to be located in [001] plane. The easiest slip plane, which is $\{\bar{1}10\}$, is perpendicular to the dislocation dipole plane. In this geometric setup, x = [$\bar{1}$10], y = [001], and z = [110]. The dipole dislocations are each arranged in the middle and in the edge of the supercell along x. The purpose of this arrangement is to avoid easy glide and annihilation of the dislocation dipole (because of the planar core). Further details of the atomistic model used and the computational methodology employed can be found in the Methods section presented towards the end of the manuscript. The initial atomic structure of the dislocation is created by taking the atomic displacements from linear elasticity using anisotropic elastic theory employing the Stroh solution^9^. Additional treatment of the displacements is carried out to take into account the periodic image effects in dislocation modelling^18^.

Introducing the dislocation dipole introduces a plastic strain of

1 $${\epsilon }_{ij}=\frac{{b}_{i}{A}_{j}+{b}_{j}{A}_{i}}{2S}$$

where *A* is the dipole cut vector and *S* is the area of the simulation supercell perpendicular to the dislocation line^18–20^. To counter this strain, a homogeneous strain of opposite sign and equal to the plastic strain in magnitude is applied to the periodic supercell. In this way, the total stress of the supercell is close to zero. In the later part of this section, we will discuss the effect associated with not applying the homogeneous strain to correct the plastic strain. In the MgO case studied here, an engineering shear strain *γ*_*yz*_ of 1.77% is applied.

**Figure 1:**
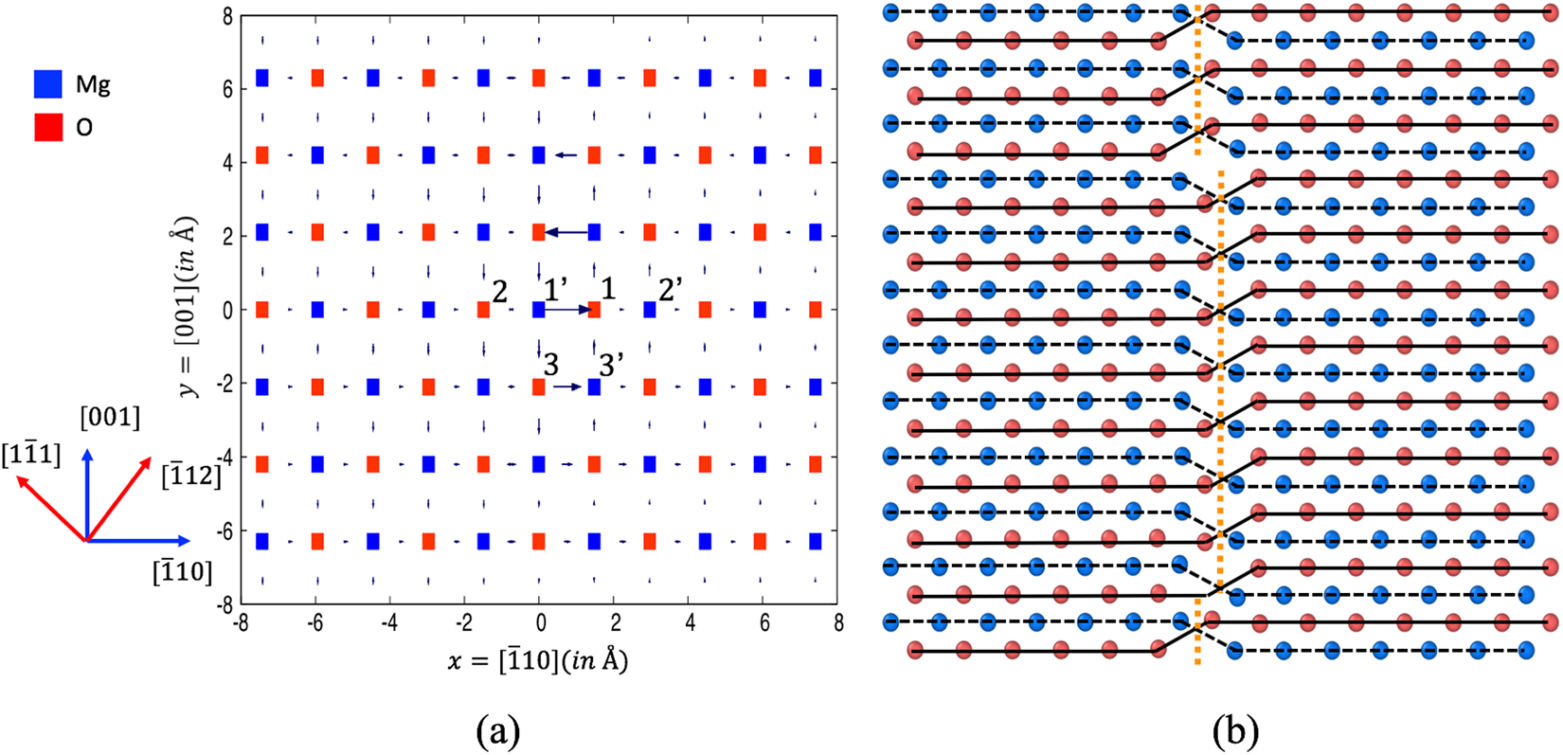
(**a**) The differential displacement map of the relaxed atomic coordinates through energy minimization for a screw dislocation in MgO. The x direction is $[\bar{1}\mathrm{10]}$, the y direction is [001] and the screw dislocation line direction is [110]. Selected vacancy sites near the dislocation core are labeled: 1, 2, 3 for V_*O*_(1), V_*O*_(2), V_*O*_(3); 1’, 2’, and 3’ for V_*Mg*_(1), V_*Mg*_(2), and V_*Mg*_(3). (see text for details). The blue axes are for “configuration 1” and the red axes for “configuration 2” (see text). (**b**) A typical dislocation jog pair as observed in MD simulations of the MgO screw dislocation containing one O vacancy. The view is along the [001] direction. The horizontal direction is $[\bar{1}10]$ and the vertical direction is [110]. The dislocation line position is marked with dashed orange lines. The layer of atoms plotted is the plane where the maximum differential displacement occurs. The blue spheres are Mg atoms and the red spheres are O atoms.

Once the dislocation geometry is set up, we can proceed with atomistic simulations. We use the Buckingham type pair potential from Lewis and Catlow^21^, which is a commonly used and reliable empirical potential for this material. Figure 1a shows the differential displacement map of the relaxed atomic coordinates through energy minimization. For the sake of clarity, only one of the two dislocations from the dipole is shown. The differential displacement map only indicates the displacement along the Burgers vector (along the dislocation line, or, in this case, the z direction). From Fig. 1a, the displacement of atoms in the core region of the screw dislocation is found to spread in the $\{\bar{1}10\}$ plane, similar to the observation of previous work^17^.

Near the core region, we chose three O sites and three Mg sites to consider as the sites for an O or Mg vacancy, as highlighted in Fig. 1a: V_*O*_(1), V_*O*_(2), V_*O*_(3), V_*Mg*_(1), V_*Mg*_(2), and V_*Mg*_(3). Table 1 lists the resulting vacancy segregation energies to the dislocation core sites, or the energy differences of putting a single vacancy in the core region and putting the vacancy in the bulk-like region (far away from the dislocations). The segregation energies range from about −0.1 to −1.1 eV, depending on the site. It is interesting to see that the lowest energy sites are sites 3 for both O and Mg cases considering that they are slightly off the center of the core. We note that, for these segregation energies, the structure of the vacancy is still localized to the original ionic site; no dissociation has yet occurred.

**Table 1:** The results of MD simulations for a screw dislocation containing a vacancy in MgO.

Vacancy site	E_*seg*_ (eV)	jog observed after MD run		energy change (eV)	Δ*E*_0_(*eV*)
		configuration 1	configuration 2		
V_*O*_(1)	−1.03	yes	—	0.02	0.11
V_*O*_(2)	−0.09	yes	—	−0.84	0.18
V_*O*_(3)	−1.12	no	yes	0.03	0.03
V_*Mg*_(1)	−1.07	yes	—	0.09	0.12
V_*Mg*_(2)	−0.05	yes	—	−0.89	0.17
V_*Mg*_(3)	−1.10	–	yes	0.07	0.07

E_*seg*_ is the segreation energy. “Energy change” denotes the change of the total energies between a localized vacancy configuration and dissociated vacancy configuration. Δ*E*_0_ is the energy difference between a dissociated vacancy configuration and the lowest energy among the localized vacancy configurations.

Our previous work^14^ found that, when such structures were heated, the vacancies dissociated. To induce such structures in our simulation cells, we subject each vacancy configuration to a thermal anneal of 1000 K for 10 ps using molecular dynamics (MD). At the end of the thermal anneal, the system is quenched to zero K, and then subjected to energy minimization. The structure is then analyzed using the DXA algorithm^22^ as implemented in Ovito^23^. The results are summarized in Table 1, with the label “configuration 1”. In the geometric setup mentioned above, after the MD treatment, the dislocation configuration containing a vacancy at site 1 or 2 exhibits dislocation jogs. However, the dislocation  containing a vacancy at site 3 does not develop  any jog or kink. That is, it remains localized.

To avoid any limitation which might be due to the geometric effect of dipole dislocations in a periodic setup, we have also used an alternate geometrical configuration, i.e., x = [$\bar{1}$12], y = [1$\bar{1}$1], and z = [110] (see Fig. 1a) labeled “configuration 2” in Table 1, to study the behavior of a vacancy at site 3. Indeed, in this different geometric setup, the dislocation jogs are observed for both the O and Mg cases after annealing, indicating that the vacancy in this configuration dissociated. A typical dislocation jog-pair, as observed after the MD simulation where the screw dislocation contains an oxygen vacancy, is shown in Fig. 1b with atomistic detail. The jog step height is *a*_0_/4[$\bar{1}$10] or one half of the Burgers vector length. We note that in Ovito visualization^23^, since only the fcc lattice of one species such as O or Mg is shown, the jog height is mistakenly amplified by a factor of two, to be *a*_0_/2[$\bar{1}$10], or full Burgers vector length. This result will have an impact on the elasticity modeling in the later part of the paper.

The total energy change after jog-pair formation (or, alternatively, vacancy dissociation) varies from site to site, ranging between −0.89 eV and 0.09 eV. This is denoted by “energy change” in Table 1. However, compared to the lowest energy site (before the jog formation), the relative energies of the dissociated structure, Δ*E*_0_, which is the energy difference between a dissociated vacancy configuration and the lowest energy among the localized vacancy configurations, after the jog formation at different sites are fairly close to each other, 0.03–0.18 eV higher. Given the small energy penalty in the jog formation, it is suggested that the jog formation events in the MD simulations are driven by entropy, not by a reduction in enthalpy. Further, the small energy difference indicates that the dislocation jogs formed at different sites are similar to each other. There are two possible reasons contributing to this small difference: one is due to the different atomic sites, and the other is due to different segment lengths between the jogs. We will see later that the latter factor is a rather weak one. The entropy contributions due to the jog formation, neither configurational nor vibrational, have been directly computed.

During the present work, we find that proper treatment of the stress state of the computational supercell plays an important role in the atomistic modeling study of jog formation. In our previous work^14^, the correction to the plastic strain during the introduction of the dislocation dipole in the supercell was not applied. We will consider the effect here. Given a screw dislocation with two jog pairs of separation length *w*, and jog height *h*, from the Peach-Koehler force on dislocations,

2 $${\bf{f}}=(\sigma \cdot {\bf{b}})\times \xi $$

where *σ* is the stress, **b** is the Burgers vector, and $\xi$ is a unit vector along the dislocation line direction. The energy change due to the stress is then, in scalar form,

3 $${\rm{\Delta }}E=\sigma bwh$$

The energy in Eq. (3) is conventionally termed “stress migration” energy since it represents the work done (and thus the energy reduction) by stress when the dislocation segment moves during the jog formation process.

**Figure 2:**
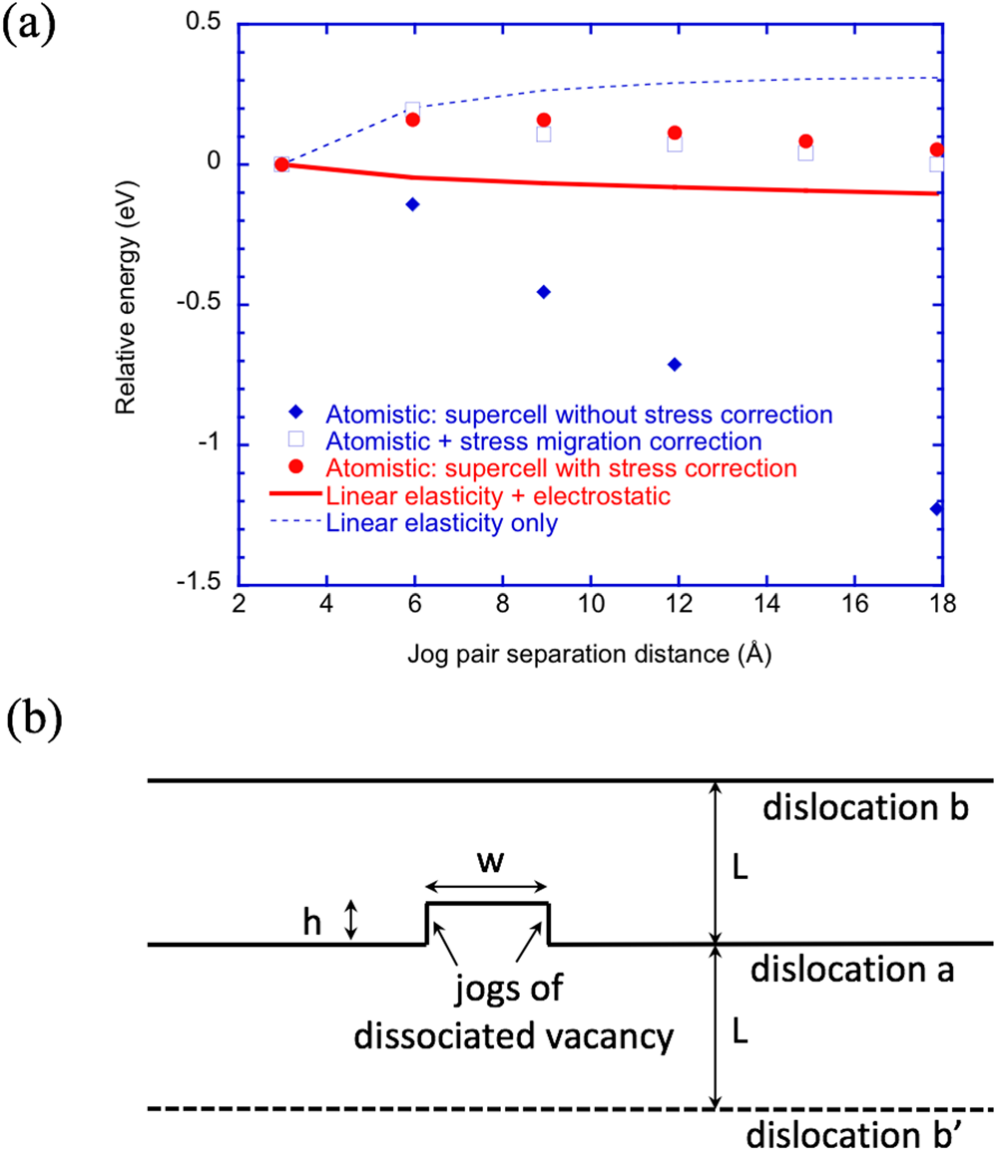
(**a**) The calculated atomistic energies of the dissociated vacancy as a function of separation of the two jogs in MgO (blue filled diamond symbols - supercell without stress correction; red filled sphere symbols - supercell with stress correction; blue square symbols - the case of supercell without stress correction plus stress migration energies). The continuous line corresponds to a model with electrostatics (charged jogs) while the dotted-line corresponds to model results without electrostatic contributions (neutral jogs). (**b**) Schematic of the dislocation model used to calculate the energetics of the dissociated vacancy as a function of jog-jog separation *w*.

Here, we reexamine the calculations originally described in our previous work^14^. In that work, the energetics of the dissociated vacancy, as a function of dissociated segment length, are determined by manual construction, in which a given number of O ions are displaced by an appropriate distance and subsequently minimized to create the separated jog structure. We repeat that calculation here with and without the stress correction to the computational supercell as described above. For the atomistic results obtained from the supercell without the stress correction, the stress migration energy calculated from Eq. (3) is also added, and compared to the atomistic results obtained from the supercell with the stress correction. The calculated atomistic energies of the dissociated vacancy as a function of separation of the two jogs in MgO are shown in Fig. 2a. The direct atomistic result *without* stress correction in the system shows a monotonic and nearly linearly decreasing behavior, resulting in an energy decrease of approximately −1.2 eV when the separation is about 18 Å compared to when the separation is only one nearest neighbor distance, or 2.97 Å, which is the same as the atomistic result of our previous work^14^. In contrast, when the energy change due to the stress migration from Eq. (3) is added to the above atomistic simulation result (with the stress level determined from atomistic simulations, approximately on the order of 2.7 GPa), the corrected result is not monotonic anymore (see Fig. 2a), and the energy is substantially shifted upward depending on the jog separation distance. This corrected result is also in good agreement with the atomistic simulation result with stress correction in the computational supercell, as shown in Fig. 2a. Thus, the significant reduction in energy observed in the original calculations is a consequence of the stress in the cell. Once corrected for, the change in energy as a function of jog separation is relatively small.

### Elasticity modeling of MgO screw dislocations

In our previous work on dissociated vacancies^14^, we derived an analytical model of the energetics of the dissociated vacancy based on both elasticity theory and electrostatics. In this subsection, we revisit that model. We use linear elasticity theory of dislocations^9^ to derive the dislocation-jog interactions and compare with the atomistic results in MgO. The elasticity model is schematically illustrated in Fig. 2b, and similar to our previous work^14^. A segment of dislocation *a* climbed a height of *h*, to form a jog-pair of width *w*. In the periodic dipole supercell, the distance between the screw dislocation dipole (*a* and *b* in Fig. 2b) is *L*.

We re-derived the elastic interactions in the elasticity model based on the explicit expression of elastic interaction energies of parallel dislocation segments as described by Hirth and Lothe^9^. The model is a modified version of the model that appears in ref.^14^.

The total energy of the system is given by

4 $$E(L,w,h,\mu ,b,\nu ,q,\epsilon )={E}_{{\inf }}^{a}+{E}_{seg}^{a}-{E}^{ab}+{E}_{electrostatic}+{E}_{self}^{jog}$$

where ${E}_{{\inf }}^{a}$ is the interaction energy between the infinite segments of dislocation *a* and the jog segment that is parallel to dislocation *a*; ${E}_{seg}^{a}$ is the interaction energy between the jog parallel segments; *E*^*ab*^ is the sum of the energies of interaction between the jog with the dislocation *b* and its periodic image *b*′, and the additional periodic images of dislocations, and is given by,

5 $$\begin{array}{rcl}{E}^{ab} & = & \sum _{n=1}^{\infty }\,{E}_{{\inf }}^{ab}(nL+h)+{E}_{{\inf }}^{ab}(nL-h)-2{E}_{{\inf }}^{ab}(nL)\\  &  & +\,{E}_{seg}^{ab}(nL+h)+{E}_{seg}^{ab}(nL-h)-2{E}_{seg}^{ab}(nL)\end{array}$$

*E*_*electrostatic*_ is the term due to the electrostatic repulsion of the two jogs, and the last term ${E}_{self}^{jog}$ describes the self energy of the jog formation (two parallel extra segments of the jog). In practice, n is summed up to 10^6^ numerically, with well converged results. The actual forms of these interaction energies are:

6 $${E}_{{\inf }}^{a}=\frac{{b}^{2}\mu }{2\pi }[\sqrt{{w}^{2}+{h}^{2}}-w-h+w\,{log}\,\frac{2w}{w+\sqrt{{w}^{2}+{h}^{2}}}]$$

7 $${E}_{seg}^{a}=-\,\frac{{b}^{2}\mu }{2\pi (1-\nu )}[w-\sqrt{{w}^{2}+{h}^{2}}+h\,{log}\,\frac{h+\sqrt{{w}^{2}+{h}^{2}}}{w}]$$

8 $${E}_{{\inf }}^{ab}(D)=\frac{{b}^{2}\mu }{2\pi }[\sqrt{{w}^{2}+{D}^{2}}-D-w\,{log}(\sqrt{{w}^{2}+{D}^{2}}+w)]$$

9 $${E}_{seg}^{ab}=\frac{{b}^{2}\mu }{4\pi }[2D-2\sqrt{{w}^{2}+{D}^{2}}+w\,{log}\,\frac{\sqrt{{w}^{2}+{D}^{2}}+w}{\sqrt{{w}^{2}+{D}^{2}}-w}]$$

10 $${E}_{electrostatic}=\frac{(q+{q}_{0})\,(q-{q}_{0})}{4\pi {\epsilon }_{0}\epsilon }\frac{1}{w}$$

11 $${E}_{self}^{jog}=\frac{{b}^{2}\mu h}{2\pi (1-\nu )}\,\mathrm{log}\,\frac{h}{e\rho }$$

In Eqs (8) and (9), *D* can be *nL* + *h*, *nL* − *h*, or *nL* depending on which term in Eq. (5) is calculated. In Eq. (10), *q* is half the charge of the full formal charge of the vacancy (1*e* for MgO) while *q*_0_ and −*q*_0_ are inherent charges for the jog pairs (taken as 0.5*e* for the MgO case^9^). Finally, in Eq. (11), $\rho$ is $\tfrac{b}{2\alpha }$ where *α* is typically around 3–5 for ionic crystals^9^. In the above equations, *μ* is the shear modulus, *ν* is the Poisson’s ratio, $\epsilon$ is the relative permittivity of the material, and ${\epsilon }_{0}$ is the permittivity of free space. The parameters used to describe MgO and UO_2_ are listed in Table 2.

**Table 2:** Parameters describing the interaction of jog pairs along a screw dislocation for MgO and UO_2_.

	*a*_0_(*Å*)	*h*(*Å*)	*b*(*Å*)	*μ*(GPa)	*ν*	${\boldsymbol{\epsilon }}$
MgO	4.212	1.49	2.978	144.3	0.29	7.3
UO_2_	5.447	2.72	3.85	78.5	0.27	10.2

*a*_0_ is lattice constant. *h* is jog height. *b* is the length of Burgers vector. *μ* is shear modulus. *ν* is Poisson’s ratio. $\epsilon$ is the relative permittivity of the material. *μ*, *ν*, and $\epsilon$ are calculated using GULP^35^.

There are a few changes compared to the old model in our previous work^14^. The most important change is the addition of the interaction energy between the jog with the periodic image of dislocation *b*, *b*′. Addition of this term changes the modeling result in a substantial way. Briefly, this interaction cancels most of the energy contribution from the interaction energy between the jog and dislocation *b*. Secondly, there is a minor change of a factor of 2 in Eq. (9), however, this term turns out to not contribute to the total energy in a significant way (after the first change of the model is applied). Thirdly, the inherent charges for the jog pairs^9^ *q*_0_ are brought into the Coulombic expression to make it more general. Finally, the self energies of the jog formation via two parallel extra segments of the jog pair are described to obtain the total energy change during the vacancy dissociation process.

Using the above elasticity model, the total energy due to the dislocation-jog interactions and the jog self formation is calculated. The parameter *α* in the self energy expression is not exactly defined or calculated from other sources. Indeed, the self energy term is only approximately correct since the expression in Eq. (11) is derived from an isolated segment of straight dislocation^9^ while in the jog case here, corner effects would also contribute. For MgO, a value for *α* of 5.6 leads to the elasticity model prediction of the jog formation energy ranging from 0.10 eV to −0.05 eV depending on the jog width (3.0 Å–33 Å). This is in good agreement with the atomistic modeling result (taking the lowest formation energy value of 0.03 eV from Table 1), with energy difference <0.1 eV.

Again, we focus on the comparison of the elasticity model with the atomistic simulations results on the dissociated O vacancy by manual construction as a function of the jog-jog separation in MgO. The advantage of this comparison is that the elasticity energy differences among configurations with different jog-jog separation distance are independent of the jog self energy. The result of this comparision is shown in Fig. 2a. A reasonable agreement between the elasticity model (including the electrostatic contribution) and the atomistic results (with stress correction) is reached. It is also clear from Fig. 2a that the elasticity model without the electrostatic contribution gives qualitatively different behavior, thereby suggesting that the long-range Coulombic repulsion helps to drive the dissociation of the vacancy leading to jog formation, which agrees with previous work^14^. In particular, without electrostatics, the model predicts that the jog-jog energy increases with separation, indicating an attraction, while the electrostatic model, after an initial nucleation barrier, predicts an energy that decreases with separation, a result of electrostatic repulsion once the jogs are separated by a significant distance.

### UO_2_ screw dislocations

Finally, to understand the generality of the dissociated vacancy mechanism, we consider the case of a screw dislocation in UO_2_. UO_2_ is the most commonly used fuel in light-water nuclear reactors. Further, UO_2_ is isostructural with other important fluorite-structured oxides, including PuO_2_, ThO_2_, and CeO_2_, the latter being an important material for the study of solid oxide fuel cells and oxygen transport. Thus, the behavior of defects at dislocations in CeO_2_ has received significant attention^7^.

UO_2_ has the calcium fluoride (CaF_2_) structure with U ions on the fcc lattice while O ions surround U ions forming a cubic structure. Screw dislocations in UO_2_ have been relatively less well studied compared to edge dislocations. Based on the Peierls-Nabarro modeling and interatomic potentials, Skelton and Walker^24^ calculated the Peierls stresses for screw dislocations gliding on {100}, {110} and {111}, and concluded that screw dislocations in UO_2_ have a preferred slip system of a_0_/2〈1 1 0〉{100}, followed by a_0_/2〈1 1 0〉{111}, similar to what is found for edge dislocations^25^, and in general agreement with experiments^26^. This finding is validated most recently by direct Peierls stress calculations using a variable charge many-body empirical potential (the second moment tight-binding potential with charge equilibration (SMTB-Q)^27^) although the magnitude of the Peierls stress differed in the two treatments.

The screw dislocations in UO_2_ are modeled in a dipole configuration with the geometric setup of the simulation cell of x = [001], y = [1$\bar{1}$0], and z = [110]. The dislocation lines are arranged to be located in the [1$\bar{1}$0] plane. We use the Morelon potential^28^ for UO_2_ for modeling of dislocations, following other works^25,29^. In the Morelon potential, the charge is −1.613*e* for O and 3.227*e* for U ions.

**Figure 3:**
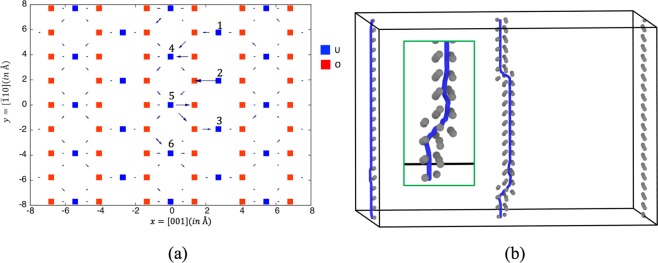
(**a**) The differencial displacement map of the relaxed atomic coordinates through energy minimization for a screw dislocation in UO_2_. The x direction is [001], the y direction is $[\bar{1}10]$, and the screw dislocation line direction (parallel to z) is [110]. Selected vacancy sites near the dislocation core, analyzed in the text, are labeled. (**b**) A typical dislocation jog pair as observed in the MD simulations of a U vacancy on the screw dislocation (in the middle of the figure). The structure was determined by the DXA algorithm^22^ as implemented in Ovito and the view is skewed slightly to emphasize the jog structure. The dislocation lines are indicated with thick blue lines. The grey atoms are U atoms not in fcc, bcc, or hcp coordination environments. The fcc lattice U atoms are hidden for clarity. Inset: an enlarged view of the bottom jog of the jog pair.

In Fig. 3a, the differential displacement map of the relaxed atomic coordinates through energy minimization is shown. From Fig. 3a, the displacement of atoms in the core region of the screw dislocation is found to be non-planar, which is different than in the case of MgO. Detailed disregistry analysis (not shown) suggests that the screw dislocation has a center on the O atom. Near the core region, we choose six U sites to consider as the creation sites for a U vacancy, labeled 1–6 in Fig. 3a. In Table 3, the vacancy segregation energy to the dislocation core sites for a localized vacancy structure are listed. They range from −0.49 to −1.14 eV. For site 5, a spontaneous relaxation (without heating) into a jog structure is obtained. However, the jog structure obtained is a metastable state since, upon heating, it further relaxes into a lower energy configuration. Therefore, we use the next lowest energy site, site 4, as the reference site for subsequent energy comparisons.

**Table 3:** The results of MD simulations for a uranium vacancy on a screw dislocation in UO_2_.

U vacancy site	E_*seg*_ (eV)	Jog observed after MD run	Energy change (eV)	Δ*E*_0_(*eV*)
1	−0.81	yes	−0.78	−0.46
2	−0.90	yes	−0.54	−0.32
3	−0.83	yes	−0.85	−0.55
4	−1.12	yes	−0.61	−0.61
5	−1.14^*a*^	yes	−0.56	−0.58
6	−0.49	yes	−1.09	−0.46

^*a*^metastable jog.

E_*seg*_ is the segregation energy. “Energy change” denotes the change of the total energies between a localized vacancy configuration and dissociated vacancy configuration. Δ*E*_0_ is the energy difference between a dissociated vacancy configuration and the lowest energy among the localized vacancy configurations.

As for MgO, to induce potential vacancy dissociation, MD simulations at 1000 K are applied to the system for 60 ps followed by additional MD simulations at 300 K for 1 ps to allow  the system to escape any metastable configuration. After that, the system is quenched to 0 K and energy minimized. After the thermal anneal, all six configurations exhibit vacancy dissociation. Compared to site 4 (before the jog formation), the energy levels after the jog formation at different sites are substantially lower, ranging from −0.32 to −0.61 eV. The possible reason for the energy range can be due to the site-specific configurational influence to the jog geometries. The atomistic results here suggest that the mechanism of vacancy dissociation through jog formation is not limited to rocksalt-structured materials, and can be extended to more complicated lattices such as UO_2_. In fact, the mechanism is more likely in a material such as UO_2_. In this case, the jog formation events are driven by a reduction in energy in addition to the entropic contribution. In Fig. 3b, a typical dissociated vacancy structure as observed in the MD simulations is shown, both with atomistic details and the structure of the dislocation as determined with DXA highlighted. For UO_2_, the Ovito^23^ visualization captures the jog height in an approximately correct way, with the value of *a*_0_/2, as listed in Table 2. In UO_2_, the jog pair has a more complicated jog structure than in MgO, as shown in Fig. 3b.

Again, using the above elasticity model, the total energy due to the dislocation-jog interactions and the jog self formation is calculated. By setting *α* in the self energy expression to be 3.5, the prediction of the elasticity model of the energy changes due to the jog formation/vacancy dissociation on the screw dislocation is in close agreement with the atomistic modeling results, ranging from −0.53 eV to −0.67 eV depending on the jog pair separation width (2.7 Å–30 Å). This suggests that the total energy dependence on the jog pair separation width is quite weak. Taking the largest energy change due to the jog formation (−0.61 eV) in the atomistic modeling results, the elasticity model prediction is considered to be in fairly good agreement with our atomistic modeling results.

One of the major implications of this mechanism, of the dissociation of vacancies into jog-pairs on screw dislocations, is the possibility of this mechanism occurring at low-angle twist grain boundaries, as demonstrated in the previous work on ionic rocksalt compounds^14^. The current work investigated the mechanism more systematically and corrected some limitations of the previous study^14^. In the previous study^14^, the conclusions regarding the dissociation of vacancies into jog-pairs at low angle twist grain boundaries, which can be viewed as a network of screw dislocations, in rock-salt oxides are still correct. However, the interpretation of the results needs to be revised. The large reduction in the energy of the dissociated vacancy as one of the jogs gets closer to the adjacent misfit dislocation intersections (MDI) within the grain boundaries should be interpreted as the result of the jog-MDI interaction since the interaction between jogs and screw dislocations in MgO, as revealed in current study, is a weak interaction, and the formation of dissociated vacancy in the form of jog pairs is driven mainly by entropy. In general, the current work offers a more accurate understanding of the physical processes involved.

From the above results, it is also clear that the long-range Coulombic interaction between the jogs, while aiding in their formation, barely counters the attractive elastic force between the jog pairs. This is reflected in the conclusion from the elasticity analysis that, for both MgO and UO_2_, the dependence of the total energy change on the jog pair separation width during the vacancy dissociation is fairly weak. In the case of MgO, this is validated by the atomistic modeling result as well.

One issue to address is the validity of the empirical potentials used to describe the dislocations in the aforementioned materials. In MgO, the Buckingham type potential’s description of the dislocations was compared to DFT by Carrez *et al*.^17^. There, the atomic disregistries along two orthogonal ([001] and [1$\bar{1}$0]) directions around the core of a 〈110〉 screw dislocation in MgO were obtained from calculations using both the Buckingham potential and DFT. The agreement between the two types of simulations is excellent at zero external pressure, validating the choice of the pairwise potentials. In addition, it is shown in this paper that the calculated generalized stacking fault energy using the potential almost overlaps with the result from DFT at zero external pressure. Such agreement suggests that, at ambient pressure, the dislocation behavior in MgO can be modelled with the Buckingham potential with reasonable fidelity. In UO_2_, there are many different empirical potentials available and care must be taken for the choice of empirical potential to use for dislocation modeling. Murphy *et al*.^29^ studied the line energies and core structures of edge and screw dislocations in UO_2_ using 15 different empirical potentials and concluded that the Morelon potential provided the lowest dislocation line energies for edge and screw dislocations in UO_2_, consistent with its prediction of ordered dislocation core structures. Arguably, the Morelon potential is among the best available UO_2_ empirical potentials^30^ to model dislocations in UO_2_. While DFT modeling of dislocation core structures in UO_2_ is not available due to computational cost, we spot checked some of the dissociated vacancies jog-pair structures on screw dislocations in UO_2_ using the SMTB-Q tight-binding model^27,31^ and found that there is almost no difference in the final relaxed configurations compared to those using the Morelon potential. That is, using the SMTB-Q model we find the dissociated vacancy structure to be a local minimum in the potential energy landscape, indicating that the result using the Morelon potential is not an artifact of the potential.

It has been shown before^32^ that, under a high electric field, oxygen vacancies can be polarized in alkaline-earth-metal binary oxides such as MgO. The possible effect of highly polarizable oxygen vacancies under electrical fields on our analysis remains an open question and is beyond the scope of this study.

In conclusion, a systematic study of vacancy dissociation at screw dislocations in MgO is presented. The important role of stress migration in the atomistic modeling is identified, which corrects earlier results^14^. A reasonable agreement between the elasticity model including the electrostatic contribution and the atomistic results with stress correction is reached. Given the small energy penalty in the jog formation, it is suggested that the jog formation events in the MD simulations are driven mainly by entropy, a consequence of the almost identical cancellation of elastic attraction by electrostatic repulsion. Both atomistic study and elasticity analysis also suggest that the mechanism of vacancy dissociation through jog formation is not limited to rocksalt-structured materials, and can be extended to more complicated lattices such as UO_2_. In fact, the mechanism is more likely in a material such as UO_2_. In this case, the jog formation events are driven by a reduction in energy in addition to the entropic contribution.

## Methods

For MgO, two configurations are modelled. “Configuration 1” has a supercell along x = [$\bar{1}$10], y = [001], and z = [110], replicating 28 × 20 × 20 periods of the unitcell along x, y, and z. The supercell has dimensions of 83 *Å* × 84 *Å* × 59 *Å*, containing a total of 44800 atoms. “Configuration 2” has a supercell oriented along x = [$\bar{1}$12], y = [1$\bar{1}$1], and z = [110], replicating 16 × 12 × 20 periods of the unitcell along x, y, and z. The supercell has dimensions of 82 *Å* × 87 *Å* × 59 *Å*, containing a total of 46080 atoms. For UO_2_, the supercell in the atomistic simulations has an orientation of x = [001], y = [1$\bar{1}$0], and z = [110], replicating 20 × 12 × 10 periods of the unitcell along x, y, and z. The supercell has dimensions of 109 *Å* × 92 *Å* × 77 *Å*, containing a total of 57600 atoms.

We employ the parallel MD code LAMMPS^33^ for the simulations, and the particle-particle particle-mesh solver (pppm) as implemented in LAMMPS is used to compute the long-range Coulombic interactions. For the MgO potential used^21^, a cutoff of 8 *Å* is used. In the UO_2_ Morelon empirical potential^28^, a four-ranges potential, is used for O-O interactions, where

12 $${V}_{ij}={a}_{ij}{\exp }(\,-\,r/{b}_{ij})\,(r\le 1.2\mathop{A}\limits^{\circ })$$

13 $${V}_{ij}={b}_{0}+{b}_{1}r+{b}_{2}{r}^{2}+{b}_{3}{r}^{3}+{b}_{4}{r}^{4}+{b}_{5}{r}^{5}(1.2\mathop{A}\limits^{\circ } < r\le 2.1\mathop{A}\limits^{\circ })$$

14 $${V}_{ij}={a}_{0}+{a}_{1}r+{a}_{2}{r}^{2}+{a}_{3}{r}^{3}(2.1\mathop{A}\limits^{\circ } < r\le 2.6\mathop{A}\limits^{\circ })$$

15 $${V}_{ij}=-\,{c}_{ij}/{r}^{6}(r > 2.6)$$

A tabular form of the O-O interaction is used. The coefficients used in the O-O interactions are: *a*_*ij*_ = 11272.6, *b*_*ij*_ = 0.1363, *c*_*ij*_ = 134.0, *a*_0_ = 42.891698, *a*_1_ = −55.496542, *a*_2_ = 23.077359, *a*_3_ = −3.131396, *b*_0_ = 479.955282, *b*_1_ = −1372.530359, *b*_2_ = 1562.223303, *b*_3_ = −881.968518, *b*_4_ = 246.434690, *b*_5_ = −27.244725. The numbers given here are in more numerical precision than the parameters published in ref.^34^, however, found necessary to construct the potential accurately. In addition, a cutoff radius of 12.001 *Å* is used for the Morelon empirical potential^28^.

The canonical NVT (constant volume and temperature) ensemble is used for the simulations. The temperature control is performed by the Langevin method as implemented in LAMMPS^33^. Conjugate gradient algorithm is used for the energy minimization under constant volume condition after the correction strain is applied.
